# Elements of person knowledge: Episodic recollection helps us to identify people but not to recognize their faces

**DOI:** 10.1016/j.neuropsychologia.2016.11.001

**Published:** 2016-12

**Authors:** Graham MacKenzie, David I. Donaldson

**Affiliations:** University of Stirling, UK

**Keywords:** Person identification, Face recognition, Episodic memory, Recollection

## Abstract

Faces automatically draw attention, allowing rapid assessments of personality and likely behaviour. How we respond to people is, however, highly dependent on whether we know who they are. According to face processing models person knowledge comes from an extended neural system that includes structures linked to episodic memory. Here we use scalp recorded brain signals to demonstrate the specific role of episodic memory processes during face processing. In two experiments we recorded Event-Related Potentials (ERPs) while participants made *identify*, *familiar* or *unknown* responses to famous faces. ERPs revealed neural signals previously associated with episodic recollection for *identify* but not *familiar* faces. These findings provide novel evidence suggesting that recollection is central to face processing, providing one source of person knowledge that can be used to moderate the initial impressions gleaned from the core neural system that supports face recognition.

## Highlights

• Face recognition and person identification produce dissociable neural signals.
• Person identification is associated with a recollection Event-Related Potential.
• Episodic memory contributes to person knowledge via recollection.

## 1 Introduction

When we encounter somebody our response depends on whether we know who they are. Even unknown faces contain information that provides immediate clues to a range of characteristics, from trustworthiness (Fenske et al., 2005) to likely aggressiveness (Lefevre and Lewis, 2014). When we know a person, stored representations in long term memory are also activated, providing access to knowledge that may moderate immediate impressions. Whilst early models of face processing focussed predominantly on semantic memory as the source of person knowledge (e.g., Bruce and Young, 1986), more recent neuroanatomical accounts have highlighted the additional importance of episodic memory (Gobbini and Haxby, 2007). This merger of face processing and memory models leaves open an intriguing question – in what way does episodic memory contribute to person knowledge? To address this issue we present a study of person identification using a neural marker of episodic memory. Before outlining our study, we first briefly introduce the key elements of face processing models, the retrieval processes that support episodic memory and the brain signals that can be used to study them.

The experience of recognizing a face yet being unable to identify the person is relatively common and has stimulated theories of how person identification is achieved, in both face processing (Bruce and Young, 1986) and episodic memory (Mandler, 1980) fields. Common to both classes of model is the idea that recognition and identification are supported by distinct processes. Cognitive models of face perception (e.g., Breen et al., 2000; Bruce and Young, 1986; Burton et al., 1990) converge on the view that face recognition occurs when incoming sensory information is matched with a unique memory representation, and that person identification occurs when biographical information is retrieved. Complementary neuroanatomical models (Gobbini and Haxby, 2007, Haxby et al., 2000) describe a core system involved in analysis of visual appearance (supporting recognition) and an extended system involved in retrieval of person knowledge (supporting identification). Critically, the extended system also clearly implicates episodic memory as one element of person knowledge (see Ferreira et al. (2015) and Lundstrom et al. (2005)) along with semantic representations. What face processing models do not describe is precisely how episodic memory contributes to person knowledge.

Episodic memory models describe two retrieval processes: recollection and familiarity (Mandler, 1980, Jacoby and Dallas, 1981, Tulving, 1985, Yonelinas, 1994). Recollection involves recovery of contextual information present at encoding, while familiarity simply signals previous occurrence. These two retrieval process are dissociable on several grounds, including their differential sensitivity to experimental manipulations (see Yonelinas, 2002) and different forgetting patterns (Sadeh et al., 2016). The aim of the current investigation is to ask whether episodic memory contributes to person knowledge through recollection or familiarity. Importantly, both retrieval processes have been associated with distinct brain signals. Scalp recorded Event-Related Potentials (ERPs) have been widely used to investigate the ability to discriminate between recently studied and non-studied stimuli. ERP findings provide strong evidence for dual-process models of recognition memory (Rugg and Curran, 2007). Studies using mainly lexical stimuli have identified ERP components for familiarity and recollection, the midfrontal and left parietal old/new effects, respectively. However, this standard model is challenged on two fronts from claims that the midfrontal effect actually reflects conceptual priming (Voss et al., 2010) and that recollection for unfamiliar faces elicits an anterior effect (MacKenzie and Donaldson, 2007, MacKenzie and Donaldson, 2009, Galli and Otten, 2011). Importantly, the current investigation examines memory for famous faces, which have been shown to elicit the standard left parietal effect (Nie et al., 2014). In this context ERPs provide a robust means of measuring the contribution of episodic retrieval to performance. Furthermore, the high temporal resolution of ERPs can help to dissociate phenomena thought to occur in series, such as face recognition and person identification.

Two famous face identification experiments are described below. In each experiment, a series of faces was shown to participants, who designated each one as either familiar, identified or unknown. *Familiar* faces were recognized but could not be identified, while *identify* faces elicited retrieval of person-specific information, such as the person's name or occupation. These response options are inspired by Tulving's (1985) Remember/Know procedure, in which Remember and Know responses provide indices of recollection and familiarity, respectively. The Remember/Know procedure has been used to investigate whether semantic memories have autobiographical content in behavioural studies investigating famous names (Westmacott and Moscovitch, 2003) and famous faces (Damjanovic and Hanley, 2007). Here we use a modified version of Tulving's procedure, combined with ERP measures of retrieval processing, to identify how episodic retrieval processes (recollection and/or familiarity) support face recognition. According to the Gobbini and Haxby (2007) model, episodic memory supports person identification via the extended system but not face recognition via the core system. Thus, brain signals associated with episodic retrieval processes - recollection or familiarity - should be observed only for faces that are identified and not for faces that are recognized without being identified. The critical question is which of the two brain signals linked episodic retrieval will be observed.

## 2 Experiment 1

### 2.1 Materials and methods

The experimental design and procedures conform to the principals of the Declaration of Helsinki and were approved by the University of Stirling Psychology Ethics Committee. Twenty-eight right-handed participants reported having normal or corrected-to-normal vision, and received £5 per hour. The sample size was determined by consideration of typical sample sizes for recognition memory tasks using EEG reported in the literature. Data from 8 participants were discarded due either an insufficient number of responses in one or more experimental conditions or the contamination of EEG with artifacts. Data from the remaining 20 participants (13 females) with a mean age of 21 years (range: 18–31) were used to form the grand-average ERPs reported here.

Faces were shown on a 17″ LCD monitor; stimuli were presented and behavioural data were recorded with E-Prime (Psychology Software Tools; www.pstnet.com). Participants sat on a chair approximately one meter away from the monitor, with a button box on a desk in front of them. All faces were of famous people selected to be recognizable by a cohort of undergraduate students at the University of Stirling. These famous people included actors (e.g., Jennifer Aniston, Al Pacino), musicians (e.g., Kylie, David Bowie), politicians (e.g., Hillary Clinton, Alex Salmond), television personalities (e.g., Oprah, Terry Wogan) and members of the British Royal family. The full range of identities was chosen with the aim of capturing a spectrum from well-known to lesser-known people. Facial images were sourced from an internet image search. All images were cropped of hair and set against a black background, before being resized and positioned in the centre of the display. Faces subtended a maximum horizontal visual angle of 2° and a maximum vertical visual angle of 5°.

Greyscale images of 200 unique identities were presented as stimuli across 4 blocks of 50 faces. Each face appeared in the centre of the screen for 500 msec and was followed by a blank screen, during which participants made one of three responses: *identify, familiar*, or *unknown*. Participants were instructed to make an *identify* response if they recognized a face and could retrieve unambiguous person-specific information about the person (such as their name, or the name of a character they had played, or film they had starred in) that would be sufficient to identify them. A *familiar* response was required if a face was recognized but the person could not be identified; finally, an *unknown* response was required in cases where a face was not recognized. Following an *identify* response, a visual prompt asked the participant to identify the person verbally. Any trials where participants were unable to retrieve any information associated with the face were excluded from analysis. The experimenter pressed a button to initiate the next trial. In contrast, following a *familiar* or an *unknown* response the participant's button press initiated the next trial.

EEG was recorded from 62 electrodes embedded in an elasticized cap (Neuromedical Supplies: http://www.neuro.com). Electrode positions were based on the extended International 10–20 system (Jasper, 1958). All channels were referenced to an electrode positioned between CZ and CPZ; two further electrodes were placed on the mastoid processes. Muscle activity associated with blinking and eye movements was recorded from electrodes placed above and below the left eye and on the temples. Data were recorded and analyzed using Scan 4.3 software (http://www.neuro.com). Impedances were below 5 kΩ before recording commenced. The data were band pass filtered between 0.1 and 40 Hz and sampled every 4 msec. EEG was segmented into 1100msec epochs, including a 100 msec pre-stimulus interval. Epochs were time-locked to stimulus onset rather than to participant response due to interest in access to memory representations instead of decision processes or motor preparation. Response time differences across conditions in recognition memory research are more likely to be due to decision processes than to any delay in accessing mnemonic information (Dewhurst et al., 2006). Stimulus-locked ERPs therefore permit scrutiny of how the processing of stimuli might differ and can be interpreted in light of any response time variation across experimental conditions. Blink artifacts were removed using a regression procedure (Semlitsch et al., 1986), and voltages were baseline corrected by subtracting the mean voltage from the pre-stimulus interval from each point in the epoch. Trials were excluded from averaging if drift exceeded ±50 µV (measured by the difference between the first and last data points in the epoch) or where activity in any of the EEG channels at any point during the epoch exceeded ±100 µV. Data were re-referenced off-line to recreate an average mastoid reference. Waveforms were smoothed over a 5-point kernel. To enhance the signal-to-noise ratio, a minimum of 16 artifact-free trials per condition was set as a criterion before an individual participant's data were included in grand-average ERPs.

Grand-average waveforms were quantified by computing the mean amplitude in two consecutive latency periods: from 300 to 500 msec and 500 to 800 msec. Data were initially analyzed using three-way repeated-measures ANOVA with factors of condition (*familiar/identify/unknown*), location (frontal/parietal) and hemisphere (left/right) before planned comparisons between *familiar/unknown* and *identify/familiar* were performed separately. The ANOVA model restricted electrode factors to two levels to avoid potential breaches of sphericity (see Dien and Santuzzi (2005)). The specific electrodes used for analysis were: F3, F4, P3 and P4. Only main effects and interactions involving the condition factor are of theoretical interest and therefore only these statistics will be reported. Main effects of condition were scrutinized with Bonferroni-corrected pairwise comparisons as appropriate. Any interactions with electrode factors were further investigated by comparing the results of subsidiary analyses performed on subsets of conditions/electrode factors. Finally, the size of the differences between conditions was inspected numerically to determine where the effects are maximal.^1^

The contrast between *familiar* and *unknown* waveforms should reveal neural activity associated with face recognition, whereas the contrast between *identify* and *familiar* waveforms should reveal neural activity associated with person identification. If different patterns of neural activity are observed across conditions, then the view that face recognition and person identification rely upon distinct cognitive operations will be supported. Furthermore, if the planned ERP contrasts resemble established neural correlates of episodic retrieval for famous faces, namely the midfrontal and/or left parietal old/new effects, then to our knowledge the involvement of episodic retrieval in face recognition will be clearly implied for the first time using the ERP method.

### 2.2 Behavioural results

Table 1 shows the mean proportion of faces attracting each of the three responses, along with corresponding response times. All means are reported along with 95% confidence intervals. Faces were more likely to be identified than to be familiar and unidentified; *familiar* responses were made more slowly than the other two responses.

The proportions of faces allocated to each response category were submitted to a one-way repeated-measures ANOVA, which identified a difference between the means, *F(2,38)=4.38, p=0.019, η*_*p*_^*2*^*=0.19*. Bonferroni corrected pairwise comparisons found a significant difference between the mean proportion of *familiar* and *unknown* faces (*mean difference=0.13±0.07, p=0.001, d=0.84*). Non-reliable differences were observed between *familiar* and *identify* faces (*mean difference=0.06±0.09, p=0.158, d=0.33*), and between *identify* and *unknown* faces (*mean difference =0.06±0.11, p=0.219, d=0.28*).

The analysis of response times revealed a difference between the conditions, *F(2,38)=21.49; p<0.001; η*_*p*_^*2*^*=0.53*. Bonferroni corrected pairwise comparisons found significant differences between *familiar* and *identify* faces (*mean difference=253.22±96.20 ms, p<0.001, d=1.23*), and between *familiar* and *unknown* faces (*mean difference =246.61±95.71 ms, p<0.001, d =1.21*). The difference between *identify* and *unknown* faces was non-reliable (*mean difference =6.61±84.02 ms, p<0.250, d=0.04*).

### 2.3 Electrophysiology

**Fig. 1:**
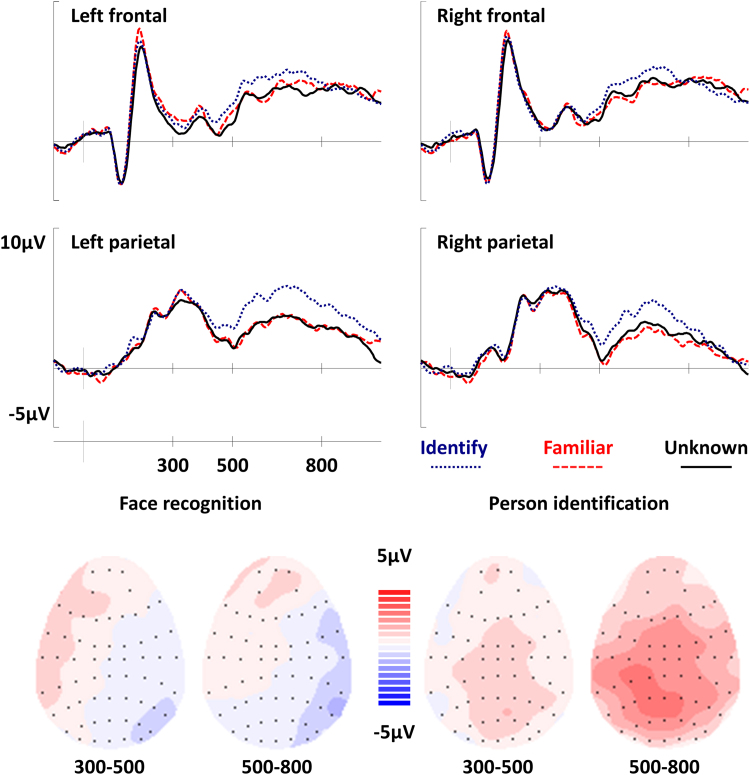
Top: Grand average ERPs for all three experimental conditions. With respect to the *familiar* condition, waveforms for the *identify* condition begin to diverge around 400 msec post-stimulus, with *identify* displaying a more positive amplitude than *familiar*. The difference between the waveforms appears to be largest at the left parietal electrode (P3). By contrast, there appears to be little difference between the *familiar* and *unknown* waveforms at the left parietal electrode. Bottom: Topographic maps showing the distribution of the ERP effects. The unknown waveform has been subtracted from the familiar waveform to show the pattern of neural activity related to face recognition, and the familiar waveform has been subtracted from the identify waveform to show neural activity associated with person identification. Scalp maps show the average neural activity during each latency period, with the front of the head at the top. Each black dot represents an electrode where the size of the difference between waveforms is known. Red colors represent areas where the difference between waveforms is positive and blue colors reflect areas where the difference is negative. The ERP effects were only robust from 300 to 500 msec for the face recognition and from 500 to 800 msec for the person identification. The effect is most pronounced at left parietal electrodes from 500 to 800 msec in the identify/familiar contrast but not in the familiar/unknown contrast.

Grand-average waveforms from all three conditions at the frontal and parietal electrodes used for analysis are shown in Fig. 1. The mean number of trials per participant contributing to ERPs was: 42 *familiar*, 50 *identify*, and 66 *unknown*. As can be seen, the most pronounced difference between the waveforms appears as a positive-going shift for the *identify* waveform from approximately 500msec post stimulus onset; this difference appears to be bigger at the parietal electrodes than at the frontal electrodes. Differences between the *familiar* and *unknown* waveforms are small and appear to be restricted to the frontal electrodes from 300 to 500 msec.

#### 2.3.1 Omnibus analysis

No differences were observed between the three waveforms during the 300-500msec latency period (see Table 2); however, the interaction between condition and hemisphere approached significance, *F(2,38)=3.22; p=0.051; η*_*p*_^*2*^*=0.14*. From 500 to 800 msec, the analysis identified a main effect of condition, *F(2,38)=8.29, p=0.001, η*_*p*_^*2*^*=0.30*. Bonferroni-corrected pairwise comparisons found that the *identify* waveform differed from both the *familiar* (*mean difference=1.62±1.18 μV, p=0.006, d=0.81*) and *unknown* (*mean difference=1.50±1.07 μV, p=0.005, d=0.82*) waveforms, but that there was no difference between the *familiar* and *unknown* waveforms (*mean difference=0.12±1.23 μV, p>0.250, d=0.06*). The condition factor did not interact with any of the electrode factors.

#### 2.3.2 Face recognition

In the 300-500msec latency period the analysis of the *familiar* and *unknown* waveforms revealed an interaction between condition and hemisphere, *F(1,19)=6.59; p=0.019; η*_*p*_^*2*^*=0.26*, reflecting a more positive amplitude for *familiar* than *unknown* on the left hemisphere but not on the right hemisphere The effect was maximal (yet not reliable) at the left frontal electrode (*mean difference=0.63±1.22 µV, t(19)=1.1, p<0.250, d=0.24*).^2^ No differences were observed between the waveforms in the 500-800msec latency period. This pattern of results provides some support for the view that familiarity for faces is associated with a left frontal effect from 300 to 500 msec.

#### 2.3.3 Person identification

The *identify* and *familiar* waveforms did not differ in the 300-500msec latency period. However, from 500 to 800 msec, the analysis revealed a main effect of condition, *F(1,19)=12.85; p=0.002, η*_*p*_^*2*^*=0.40,* representing a more positive-going waveform for *identify* than for *familiar*. The effect was maximal at the left parietal electrode (*mean difference=2.29±1.13 µV, t(19)=4.25, p<0.001, d=0.95*).

### 2.4 Interim discussion

Dissociable neural signals were observed for face recognition and person identification, implying that the two phenomena result from different processes. The face recognition effect was observed from 300 to 500 msec, whereas the person identification effect was present from 500 to 800 msec. The timing of these components implies that face recognition precedes person identification. Furthermore, the left parietal effect previously associated with episodic recollection was observed for person identification, implying that episodic memory contributes to person knowledge through recollection and not through familiarity. This interpretation rests upon the assumption that the left parietal effect reflects episodic recollection. An extensive literature reports modulations of the left parietal effect in a manner consistent with recollection (Rugg and Curran, 2007), moreover recent tests of semantic memory report left parietal effects only when semantic categories have an episodic component (Renoult et al., 2015). Taken together, the wider literature unequivocally supports the view that the left parietal effect reflects episodic recollection.

The pattern of activity associated with face recognition is harder to interpret definitively, but suggests the presence of a weak midfrontal effect. Although there was a difference between *familiar* and *unknown* waveforms across left hemisphere electrodes, the effect size was small and the difference was not significant at the electrode where the effect was biggest. Such weak evidence may stem from participants making relatively few *familiar* responses. Data from 8 participants were discarded for providing fewer than 16 trials; given the ambiguous nature of these data we conducted a second experiment using more stimuli.

## 3 Experiment 2

### 3.1 Materials and methods

The experimental design and procedures conform to the principals of the Declaration of Helsinki and were approved by the University of Stirling Psychology Ethics Committee. Twenty-six right-handed participants reported having normal or corrected-to-normal vision, and received £7.50 per hour. Data from 2 of these participants were discarded due the contamination of EEG with low-frequency artifacts, most likely resulting from sweating. Data from the remaining 24 participants (17 females) with a mean age of 22 years (range: 18–34) were used to form the grand-average ERPs reported here.

The experimental procedures and design were the same as described above for Experiment 1 except for the number and quality of the stimuli. Faces were presented in colour and set against their native backgrounds. We reasoned that greyscale presentation and cropping of external features for the stimuli used in Experiment 1 might have rendered some of the faces more difficult to recognize and that high quality colour images should be used in Experiment 2. The 400 unique identities were presented as stimuli across four blocks of 100 faces. The EEG acquisition and data processing parameters matched those described for Experiment 1.

### 3.2 Behavioural results

Table 1 shows the mean proportion of faces attracting each of the three responses, along with corresponding response times. As was the case in Experiment 1, *familiar* responses were less likely than *identify* responses, and *familiar* responses were made more slowly than the other two responses.

The proportions of faces allocated to each response category were submitted to a one-way repeated-measures ANOVA, which identified a difference between the means, *F(1.61,36.96)=6.91, p=0.005, η*_*p*_^*2*^*=0.23*. Bonferroni corrected pairwise comparisons found a significant difference between the mean proportion of *familiar* and *unknown* responses (*mean difference=0.15±0.10, p=0.003, d=0.76*). Non-reliable differences were observed between *familiar* and *identify* responses (*mean difference=0.06±0.04, p=0.187, d=0.40*), and between *identify* and *unknown* responses (*mean difference=0.09±0.13, p=0.239, d=0.37*).

The analysis of response times revealed a difference between the conditions, *F(2,46)=32.56; p<0.001; η*_*p*_^*2*^*=0.59*. Bonferroni corrected pairwise comparisons found significant differences in all three pairwise comparisons *(familiar/identify mean difference=189.49±104.22 ms, p=0.003, d=0.77; familiar/unknown mean difference=350.17±68.82 ms, p<0.001, d=2.15; identify/unknown mean difference =160.68±92.89 ms, p=0.005, d=0.73*).

#### 3.2.1 Electrophysiology

**Fig. 2:**
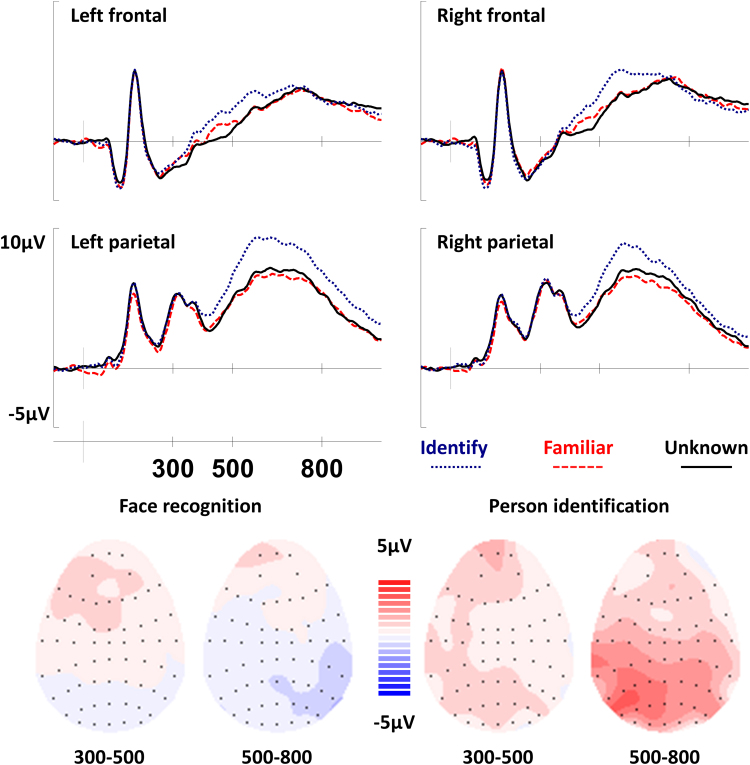
Top: Grand average ERPs for all three experimental conditions. As in Experiment 1, the difference between *identify* and *familiar* waveforms appears to be largest at the left parietal electrode. While there is little difference between the *familiar* and *unknown* conditions at parietal electrodes the waveforms clearly diverge at frontal electrodes between 300 and 500 msec. Bottom: Topographic maps showing the distribution of the ERP effects. The *unknown* waveform has been subtracted from the *familiar* waveform to show the pattern of neural activity related to face recognition, and the *familiar* waveform has been subtracted from the *identify* waveform to show neural activity associated with person identification. Scalp maps show the average neural activity during each latency period, with the front of the head at the top. Each black dot represents an electrode where the size of the difference between waveforms is known. Red colors represent areas where the difference between waveforms is positive and blue colors reflect areas where the difference is negative. The ERP effects were robust from 300 to 500 msec for the face recognition, and from 500 to 800 msec for person identification. As can be seen, the effects are most pronounced at left parietal electrodes from 500 to 800 msec in the *identify*/*familiar* contrast but not in the *familiar*/*unknown* contrast.

Fig. 2 shows ERP waveforms for all three conditions at the frontal and parietal electrodes used for analysis. The mean number of trials per participant contributing to ERPs was: 76 *familiar*, 91 *identify*, and 128 *unknown*. The most prominent feature of the waveforms is the large difference between the *identify* and *unknown* waveforms that can be observed at the left parietal electrode. This apparent left parietal effect is absent for familiar faces. Data were analyzed using the same ANOVA models and electrodes specified above for Experiment 1.

#### 3.2.2 Omnibus analysis

A main effect of condition was observed during the 300-500msec latency period, *F(2,46)=5.10; p=0.010; η*_*p*_^*2*^*=0.18*. Bonferroni-corrected pairwise comparisons found that the *identify* waveform differed from the *unknown* waveform (*mean difference=0.97±0.79 μV, p=0.013, d=0.65*) but that there were no reliable differences between the *identify* and *familiar* waveforms (*mean difference=0.68±0.90 μV, p=0.193, d=0.38*) or between the *familiar* and *unknown* waveforms (*mean difference=0.29±0.72 μV, p>0.250, d=0.21*).

From 500 to 800 msec, the analysis identified a main effect of condition, *F(2,46)=7.60, p=0.001, η*_*p*_^*2*^*=0.25*. Bonferroni-corrected pairwise comparisons found reliable differences between the *identify* and *familiar* waveforms (*mean difference=1.67±1.21 μV, p=0.008, d=0.69*) and between the *identify* and *unknown* waveforms (*mean difference=1.49±1.36 μV, p=0.029, d=0.58*) but that there was no reliable difference between the *familiar* and *unknown* waveforms (*mean difference=0.18±0.96 μV, p>0.250, d=0.10*). The ANOVA also identified interactions between condition and location, *F(2,46)=5.23, p=0.009, η*_*p*_^*2*^*=0.18*, and between condition, location and hemisphere, *F(2,46)=8.46, p=0.001, η*_*p*_^*2*^*=0.27*. See Table 3 for results from all analyses.

#### 3.2.3 Face recognition

In the 300–500 msec latency period analysis of the *familiar/unknown* waveforms revealed an interaction between condition and location, *F(1,23)=4.86; p=0.038; η*_*p*_^*2*^*=0.17*, reflecting the presence of a difference between the waveforms at the frontal location only, where the *familiar* waveform is a more positive-going than the *unknown* waveform. The effect was maximal (but only approached significance) at the left frontal electrode (mean difference =0.66±0.68 µV), *t(23)=2.0, p=0.058, d=0.41*. No differences were observed between the waveforms in the 500–800 msec latency period (see Table 3). This pattern of results indicates that familiarity for faces is associated with a frontal effect from 300 to 500 msec.

#### 3.2.4 Person identification

No differences were observed between the *identify* and *familiar* waveforms in the 300-500msec latency period. From 500 to 800 msec, however, the analysis revealed a main effect of condition, *F(1,23)=11.32; p=0.003; η*_*p*_^*2*^*=0.33*, reflecting a more positive-going waveform for *identify* faces, along with interactions between condition and location, *F(1,23)=7.13; p=0.014; η*_*p*_^*2*^ *=0.28,* and a three-way interaction between condition, location and hemisphere, *F(1,23)=7.81; p=0.010; η*_*p*_^*2*^*=0.25*. The condition by location interaction is due to the difference between the waveforms being greater at the parietal location than at the frontal location. The three way-interaction arises because the effect is bigger on the left hemisphere at the parietal location, *F(1,23)=10.85; p=0.003; η*_*p*_^*2*^*=0.32,* but not at the frontal location, *F(1,23)=0.54; p>0.250; η*_*p*_^*2*^*=0.02.* The effect was maximal at the left parietal electrode *(mean difference=2.78±1.32 µV, t(23)=4.34, p<0.001, d=0.88*).

In summary, analysis of the ERP data thus far shows that face recognition is associated with a frontal effect from 300 to 500 msec and that person identification is associated with a left parietal effect from 500 to 800 msec. The lower panel of Fig. 2 shows scalp maps depicting the distribution of the ERPs separately for face recognition and person identification averaged across the 300–500 msec and 500–800 msec latency periods used for analysis. It would appear that these frontally and parietally distributed effects reflect different cognitive operations, but this claim requires a demonstration of formal stochastic independence.

#### 3.2.5 Topographic analysis

The amplitude of the *unknown* waveform was subtracted from the *familiar* waveform, and the amplitude of the *familiar* waveform was subtracted from the *identify* waveform, on a point-by-point basis to derive subtraction waveforms representing the size of the ERP differences. These waveforms were quantified between 300 and 500 msec and 500 and 800 msec, respectively, to provide a measure of the size of the face recognition and person identification effects for all electrodes and participants. The data were then rescaled according to the max-min method described by McCarthy and Wood (1985) to minimize the possibility that gross differences in the size of the two effects would lead to spurious interactions.

Data were submitted to three-way repeated-measures ANOVA with factors of condition (*face recognition*/*person identification*), location (frontal/parietal) and hemisphere (left/right). The ANOVA revealed interactions between condition and location, *F(1,23)=7.33, p=0.013, η*_*p*_^*2*^*=0.24*, and between condition, location and hemisphere, *F(1,23)=4.66, p=0.042, η*_*p*_^*2*^*=0.17*. (The main effect of condition, *F(1,23)=0.05, p=0.824, η*_*p*_^*2*^ *<0.01*, and condition x hemisphere interaction, *F(1,23)=0.86, p=0.362, η*_*p*_^*2*^*=0.04*, were not significant.) As can be seen in Fig. 2, the significant interactions reflect the difference between the frontal distribution of the face recognition effect and the left parietal distribution of the person identification effect. The analysis therefore provides compelling evidence supporting the claim that there are differences in the underlying cognitive operations supporting familiar face recognition and person identification, which is consistent with the view that discrete neural populations generate the effects.

### 3.3 Face processing ERP components

Although our primary focus is on the ERP old/new effects, for completeness here we present a final set of analyses targeting two ERP components that are often modulated in face processing tasks, namely the N170 and N250. The N170 is a negative-going deflection of the waveform peaking around 170 msec after stimulus onset that can be observed at inferior sites over the temporal-occipital boundary; the effect can be observed bilaterally but is predominantly larger on the right hemisphere (Bentin et al., 1996). The N250 component peaks around 250msec, and can be observed at the same electrodes as the N170 (Schweinberger et al., 2002). The function of these components during face perception tasks is thought to reflect key stages in face recognition (for a review see Schweinberger and Neumann, 2016). There has been a tendency to map these ERP effects onto discrete modules described by face processing models (e.g., Bruce and Young, 1986). Accordingly, the N170 has been linked to early perceptual analysis of faces, or ‘Structural Encoding’, and the N250 with access to memory representations for faces, or ‘Face Recognition Units’.

Both the N170 and N250 components are typically analyzed with average electrode referenced ERPs rather than the average-mastoid reference used in recognition memory research. In order that we can compare our results with antecedent cases reported in the literature baseline-corrected EEG data were transformed to an average electrode reference, then smoothed and sifted of artefacts using the same parameters described above for the average mastoid-referenced data. ERPs were again formed for the three experimental conditions: *familiar*, *identify*, and *unknown*. Reprocessing led to a slightly different number of trials being rejected for artefacts in Experiment 2, and so the mean number of trials per participant contributing to ERPs was: 76 *familiar*, 88 *identify*, and 129 *unknown*. Trial numbers for Experiment 1 ERPs are the same as reported above when the average mastoid reference was used. ERPs were quantified into two latency periods associated with N170 (160–190 msec) and N250 (230–400 msec) effects in the literature (Kaufmann et al., 2009, Gosling and Eimer, 2011). Since both of these ERP effects are typically observed at inferior tempero-occipital sites bilaterally, albeit more pronounced on the right hemisphere, data were analyzed in a 3 condition (*identify/familiar/unknown*)×hemisphere (left/right) ANOVA, performed on electrodes P7 and P8.

**Fig. 3:**
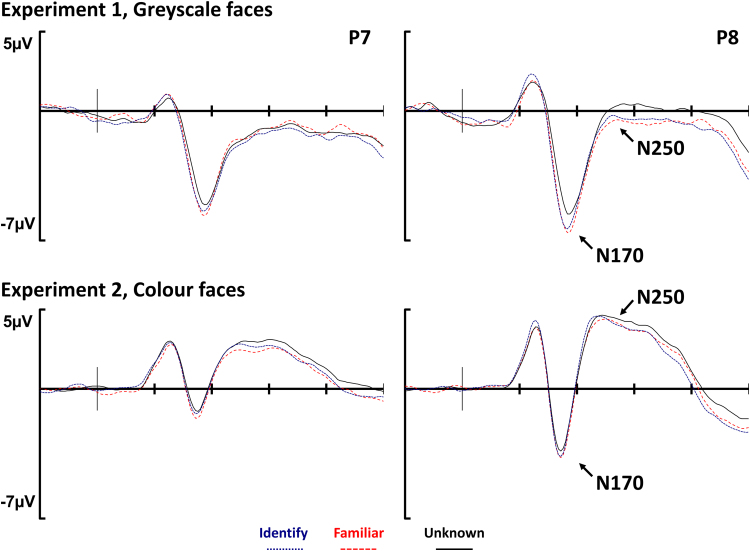
Grand average ERPs for all three experimental conditions at inferior temporal electrodes on either hemisphere (P7/8) shown for both greyscale (Experiment 1) and colour (Experiment 2) faces. On the x-axis each tick mark represents 100 msec. The N170 (160–190 msec) is more negative-going for both *familiar*/*identify* compared to *unknown* in Experiment 1, and more negative-going for *familiar* compared to *unknown* in Experiment 2. The N250 (230–400 msec) is only present on the right hemisphere for Experiment 1, where the waveform for *familiar* is more negative-going than the waveform for *unknown*. In Experiment 2, the N250 is present bilaterally, and it is more negative-going for both *familiar*/*identify* with respect to the *unknown* waveform.

Fig. 3 shows waveforms for all three conditions at left and right hemisphere inferior parietal electrodes (P7/8). The top of the figure shows data from Experiment 1 using greyscale faces; a large negative-going deflection of the waveforms can be observed clearly around 170msec at the right-hemisphere site. The amplitude of this deflection is greatest for the familiar waveform and smallest for the *unknown* waveform. The bottom of Fig. 3 shows data from Experiment 2 using colour faces. Again, an N170 is apparent with the colour faces, although the differences between the waveforms is relatively small.

#### 3.3.1 Experiment 1, Greyscale faces

A main effect of condition was observed on the N170, *F(2,38)=11.45; p<0.001; η*_*p*_^*2*^*=0.38.* The interaction between condition and hemisphere was not reliable, *F(2,38)=0.87; p=0.425; η*_*p*_^*2*^*=0.04*. Bonferroni-corrected pairwise comparisons identified reliable differences between the *familiar* and *unknown* waveforms (*mean difference*=*1.13±0.82* *μV, p*=*0.006*, *d*=*0.82*), and between the *identify* and *unknown* waveforms (*mean difference*=*1.06±0.68 μV, p*=*0.002*, *d*=*0.91*). However, no difference was observed between the *familiar* and *identify* waveforms (*mean difference*=*0.07±0.55* *μV, p>0.999, d=0.07*).

The N250 was quantified between 230 and 400 msec. Analysis found that the main effect of condition was not reliable, *F(2,38)=2.67; p=0.082; η*_*p*_^2^*=0.12.* However, there was a significant interaction between condition and hemisphere, *F(2,38)=3.36; p=0.045; η*_*p*_^*2*^*=0.15*. When data from either hemisphere were analyzed separately no effect of condition was observed on the left hemisphere, *F(2,38)=0.67; p=0.517; η*_*p*_^*2*^*=0.03,* but an effect was present on the right hemisphere, *F(2,38)=5.01; p=0.012; η*_*p*_^*2*^*=0.21.* Bonferroni-corrected pairwise comparisons found a difference between the *familiar* and *unknown* waveforms (*mean difference=0.99±0.88 μV, p=0.023, d=0.66*), but no reliable differences between waveforms for *identify* and *familiar* (*mean difference=0.21±0.70 μV, p>0.999, d=0.17*) or between waveforms for *identify* and *unknown* (*mean difference=0.78±1.00 μV, p=0.160, d=0.46*). Thus the modulation of the N250 is due to a more negative-going waveform for *familiar* than *unknown*.

#### 3.3.2 Experiment 2, Colour faces

A main effect of condition was observed on the N170, *F(2,46)=3.60; p=0.035; η*_*p*_^*2*^*=0.13*. The interaction between condition and hemisphere was not reliable, *F(2,46)=0.10; p=0.907; η*_*p*_^*2*^*=0.01*. Bonferroni-corrected pairwise comparisons found a reliable difference between the *familiar* and *unknown* waveforms (mean difference=0.44±0.36 μV, p=0.013, d=0.64), while the differences between the *identify* and *unknown* waveforms (mean difference=0.22±0.48 μV, p=0.786, d=0.24) and between the *familiar* and *identify* waveforms (mean difference=0.23±0.42μV, p=0.536, d=0.29) were not reliable.

A main effect of condition was observed on the N250, *F(2,46)=5.05, p=0.010, η*_*p*_^*2*^*=0.18.* Bonferroni-corrected pairwise comparisons found reliable differences between the waveforms for *identify* and *unknown* (*mean difference=0.45±0.41 μV, p=0.030, d=0.57*), and between the waveforms for *familiar* and *unknown* (*mean difference=0.45±0.40 μV, p=0.024, d=0.59*), but the difference between *identify* and *familiar* was not reliable (*mean difference=0.01±0.45 μV, p>0.999, d=0.01*). The interaction between condition and hemisphere was not reliable, *F(2,46)=1.61, p=0.212, η*_*p*_^*2*^*=0.06*. Thus the N250 modulation was due to more negative-going waveforms for *familiar* and *identify* compared to *unknown*; the *familiar* and *identify* waveforms did not differ from one another.

### 3.4 Summary of results

In both experiments person identification was associated with a left parietal effect while face recognition was associated with a midfrontal effect. In addition, the N170 and N250 components were modulated by familiarity, though the effects did not entirely replicate across experiments.

## 4 General discussion

Our reactions to people often depend on whether we know them; episodic and semantic memories provide distinct elements of person knowledge. But how exactly does episodic memory help us know whether somebody is friend or foe? Episodic representations can be accessed either through familiarity or recollection: we investigated which of these processes supports person identification. Participants were presented with images of famous faces and indicated whether they could *identify* the person depicted, whether the face was merely *familiar*, or whether the face was *unknown*. In two experiments, the left parietal ERP effect associated with recollection was present for person identification (as revealed by the contrast between the *identify* and *familiar* conditions), but not for face recognition (in the absence of identification; i.e., the contrast between the *familiar* and *unknown* conditions). Instead, face recognition produced a midfrontal ERP effect. Critically, however, the brain signals associated with face recognition and person identification conditions exhibited different functional, temporal and spatial characteristics. Taken together, these findings based on the topography of the observed ERP effects provide strong evidence that recollection contributes to person identification but not face recognition. Below we consider these results and their theoretical implications.

### 4.1 Person identification

The present results suggest that episodic memory contributes to person knowledge through recollection of contextual information from previous encounters with the person – clarifying episodic memory's role in face processing. When identifying Arnold Schwarzenegger, we do so by recovering information from previous episodes, such as scenes from his movies (or when and where we watched them), in addition to accessing person knowledge from semantic memory. This finding advances our understanding of the role that face processing plays in wider behavior. One key implication is that simply feeling that a person is familiar is unlikely to be sufficient to moderate one's immediate reactions; instead, retrieval of person knowledge such as the recovery of contextual details from prior episodes is required.

How strong is the evidence presented here? The ERP data themselves are extremely robust: two experiments identified clear positive-going deflections over left parietal scalp, from 500 to 800 msec. The effects strikingly resemble the left parietal old/new effect observed in episodic memory tests (e.g., Curran, 1999; MacKenzie and Donaldson, 2009; Rugg et al., 1998). There are, of course, distinct differences between the traditional memory experiments and our face processing task: the current experiment involved no recent study episodes, and therefore no new (unstudied) baseline. To our minds, however, neither of these design elements cast doubt on the brain signals seen here being left parietal effects.

Given that task performance did not require episodic memory *per se*, and could in theory be supported by semantic memory alone, it would be more parsimonious to interpret the person identification effect in terms of an N400. Waveform modulations reported as N400 effects have been observed for familiar faces and argued to reflect access to semantic representations (Wiese and Schweinberger, 2015, Schweinberger, 1996; Schweinberger and Burton, 2003). Bruce and Young (1986) distinguish between *visually-derived* and *identity-specific* semantics, referring to information that is derived from a face without the face necessarily having to be identified, and the knowledge that supports person identification, respectively. According to this viewpoint, semantic representations would necessarily have been accessed for both face recognition and person identification conditions in the present experiments – even if *identity-specific* semantic information might be retrieved more successfully for the *identify* condition. If the left parietal effect were in fact an N400, and the N400 reflects access to semantic memory, then presumably it should have been observed in both conditions, rather than exclusively for person identification. Moreover, the N400 is typically observed over central or centro-parietal midline sites, and is restricted to a 300–600 msec post-stimulus latency period. The effect we observe for person identification thus does not share the functional significance, topographic distribution or temporal profile of the N400 effect. Rather, its character resembles a classic left parietal effect associated with recollection from episodic memory.

More significant concern exists around how best to characterize the role that the left parietal effect plays in recollection. The precise functional significance of the effect remains unclear: for example, recent evidence suggests the left parietal effect is sensitive not only to the amount of recollection present, but also to the quality of information retrieved (Murray et al., 2015). In addition, recollection for unfamiliar faces has been shown to exhibit a more anteriorly distributed effect – instead of the left parietal effect (MacKenzie and Donaldson, 2007, MacKenzie and Donaldson, 2009, Galli and Otten, 2011, Nie et al., 2014), leading to the hypothesis that recollection only elicits left parietal effects when information is associated with pre-existing long-term memory representations. From this perspective, the current findings suggest that recollection of familiar and unfamiliar faces may be supported by distinct neural mechanisms – a key question for future research.

### 4.2 Face recognition

Face recognition was associated with an early (300–500 msec) ERP effect. In Experiment 1, the effect was not statistically robust and only observed on the left hemisphere in the *familiar* condition, whereas in Experiment 2, it was present at midfrontal sites in the contrast between *familiar* and *unknown* waveforms. The lack of direct replication of the effects across the two experiments limits speculation regarding its functional significance. Nonetheless, our view is that the effect observed in Experiment 2 resembles the midfrontal old/new effect (cf. Curran, 2004) associated with familiarity (Rugg et al., 1998; but see Voss and Paller, 2006). The observation of this effect in the present experiments is noteworthy given that there was no recent study history for the faces, suggesting that it is the absolute familiarity of the stimuli that produces the effect, rather than incremental familiarity due to recent study phase exposure (for discussion see Bridger et al., 2014; MacKenzie and Donaldson, 2007). More important for present purposes are the time course and functional significance of the effect. First, the frontal effect occurs before the left parietal effect, consistent with the view that faces are recognized before people are identified.

### 4.3 Theoretical implications

According to our results, some of the information contributing to person knowledge, which would typically be characterized as a form of semantic memory, is in fact recollected from episodic memory. In broad terms, these data suggest that semantic and episodic memory do not operate as entirely separate memory systems, but instead work together in the pursuit of task goals (cf. Greve et al., 2008). The distinction between episodic and semantic memory is ubiquitous within the memory literature (Squire and Zola, 1998), and the two systems are generally treated as separate entities by researchers. Recently, however, Renoult et al. (2015) have argued that episodic information can bind to semantic representations concerning identity, and that this happens when semantic representations are personally significant. The data we present here complement the findings of Renoult and colleagues, and support the view that episodic information can be yoked to famous identity representations. While Renoult *et al*. used famous names as stimuli and we used famous faces, the same multi-modal famous identity representations were accessed across studies and the same ERP component (the left parietal old/new effect, sometimes referred to as the ‘Late Posterior Complex’) was observed. These findings show that the same retrieval processes are used to probe semantic representations of famous people, regardless of the nature of the retrieval cues used (faces or names). It therefore seems likely that the recollection of episodic memory in face identification tasks is not in fact an epiphenomenon due to the particular experimental procedures that are employed, but rather reflects a core process of spontaneous retrieval occurring when famous people are represented. Here we consider famous faces as a proxy for personally familiar identities; however, it may be the case that the involvement of episodic recollection observed here is restricted to the case of faces that are familiar through the public domain. Further research on this question is warranted.

The pattern of behavioural data replicated closely across the two experiments, except for response times for faces that were endorsed as unknown. The two experiments were identical save for the quality and number of stimuli. In Experiment 1 faces were greyscale and cropped, while in Experiment 2 twice the number of faces were shown, and the faces were in full colour with background information preserved. The near identical proportions of faces allocated to each of the three responses contrasts with much faster responses for unknown faces in Experiment 2. It seems unlikely that the change in colour of the stimuli from greyscale to full colour should lead to increased response times. However, it is possible that it was easier to decide that a face could not be recognized when it was presented in colour than when it was cropped and greyscale.

A final pattern of interest in the response time data concerns the slower responses for faces that were *familiar* than for faces that were *identified*, particularly when neural activity indicated that face recognition is processed before person identification. It is common in tests of episodic memory using the Remember/Know procedure that Know responses assumed to reflect familiarity are slower than Remember responses assumed to reflect recollection; importantly, this contrasts with dual process models, which state that familiarity occurs before recollection. Dewhurst et al. (2006) explain this apparent contradiction as a consequence of longer decision processes when an item is recognized on the basis of familiarity. According to this view, participants feel that an item is familiar and wait to see if they can recollect any further information about it before making a response. The same principal likely applies in the current experiments, where participants first recognize a face and then wait to see if they can retrieve any relevant person knowledge before making a *familiar* response.

### 4.4 A note on face processing ERPs

Given the nature of the present experiments, one obvious question is whether our face recognition ERP effects simply reflect the consequences of differences in the perceptual analysis of faces, as indexed by the N170 component. Effects of face familiarity on the N170 have been reported (Caharel et al., 2014); however, the claim that the component is sensitive to familiarity is contentious: many researchers have failed to observe any modulation of the component when comparing familiar and unfamiliar faces (e.g., Schweinberger et al., 2002), or experimentally familiarized and unfamiliar faces (e.g., Tanaka et al., 2006). In the two experiments presented here N170 modulations were observed, with the component being largest for *familiar* faces. However, because we relied on life-long exposure to faces in our method we cannot be sure whether unknown faces are unfamiliar or simply familiar-but-not-recognized. As such, the modulation of the N170 cannot be attributed to changes in familiarity *per se*. Regardless, and more important for present purposes, the subsequent face recognition effects exhibited a different pattern across conditions, being largest for *identify* faces. Overall, therefore, the ERP data suggest that the face recognition and person identification effects that we observe cannot be accounted for simply by differences in the initial perceptual processing of the stimuli.

The principal reason for analyzing the N250 component was to assess the possibility than the midfrontal effect we observe for face recognition when ERPs were referenced to the average of the two mastoid channels may relate in some way to the N250, which has been described as the face memory effect (Schweinberger and Neumann, 2016). The N250, which is observed at inferior occipital-temporal sites bilaterally, although usually larger on the right hemisphere, has been shown to track with the learning history of faces such that the magnitude of the effect increases as faces become more familiar (Tanaka et al., 2006). Crucially, with an average reference, the negative-going deflections of the N250 observed at inferior temporal-occipital sites are accompanied by a positive-going effect at the frontal location. The pattern of waveform modulation observed for the N250 and the midfrontal effect is very similar, and it is therefore tempting to speculate that the two effects are in some way related. Future research seeking to investigate whether the two effects exhibit equivalent sensitivity to experimental manipulations, or can be influenced independently, is clearly warranted.

## 5 Conclusion

Using ERPs and a real-world face processing task, we have demonstrated that person identification and face recognition elicit different patterns of brain activity, providing novel evidence that these two phenomena result from dissociable processes. In two experiments, person identification elicited a left parietal ERP effect linked with episodic recollection, which suggests that one element of person knowledge comes from the recollection of contextual information from past encounters with that person. In our daily lives, the ability to distinguish between friend and foe thus depends crucially upon the operation of episodic recollection.

## Figures and Tables

**Table 1:** Behavioural results.

Experiment 1	Familiar	Identify	Unknown
Proportion	0.27±0.05	0.33±0.03	0.39±0.05
Time (msec)	1482.88±116.18	1229.66±79.49	1236.27±69.20

Experiment 2			
Proportion	0.26±0.04	0.32±0.05	0.41±0.06
Time (msec)	1411.34±117.91	1221.85±79.67	1061.17±86.29

**Table 2:** Experiment 1 ERP analysis.

	**F**	**Probability**	**Effect Size (η_p_^2^)**
**Familiar/identify/unknown 300–500 msec**			
Condition	1.14	0.331	0.06
Condition×location	0.34	0.715	0.02
Condition×hemisphere	3.22	0.051	0.14
Condition×location×hemisphere	0.64	0.531	0.03

**Familiar/identify/unknown 500–800 msec**			
*Condition	8.29	0.001	0.30
Condition×location	1.82	0.175	0.09
Condition×hemisphere	2.93	0.065	0.13
Condition×location×hemisphere	0.30	0.739	0.02

**Familiar/unknown 300–500 msec**			
Condition	0.14	0.709	< 0.01
Condition×location	0.39	0.537	0.02
*Condition×hemisphere	6.59	0.019	0.26
Condition×location×hemisphere	0.31	0.585	0.02

**Familiar/unknown 500–800 msec**			
Condition	0.06	0.802	< 0.01
Condition×location	0.44	0.515	0.02
Condition×hemisphere	3.03	0.098	0.14
Condition×location×hemisphere	0.01	0.941	< 0.01

**Identify/familiar 300–500 msec**			
Condition	1.05	0.319	0.05
Condition×location	0.56	0.463	0.03
Condition×hemisphere	0.06	0.807	< 0.01
Condition×location×hemisphere	0.34	0.566	0.02

**Identify/familiar 500–800 msec**			
*Condition	12.85	0.002	0.40
Condition×location	2.42	0.136	0.11
Condition×hemisphere	0.22	0.646	0.01
Condition×location×hemisphere	0.33	0.570	0.02

**Table 3:** Experiment 2 ERP analysis.

	**F**	**Probability**	**Effect Size (η_p_^2^)**
**Familiar/identify/unknown 300–500 msec**			
*Condition	5.10	0.010	0.18
Condition×location	2.09	0.135	0.08
Condition×hemisphere	2.32	0.110	0.09
Condition×location×hemisphere	0.87	0.426	0.04

**Familiar/identify/unknown 500–800 msec**			
*Condition	7.60	0.001	0.25
*Condition×location	5.23	0.009	0.18
Condition×hemisphere	0.78	0.433	0.03
*Condition×location×hemisphere	8.46	0.001	0.27

**Familiar/unknown 300–500 msec**			
Condition	1.12	0.302	0.05
*Condition×location	4.86	0.038	0.17
Condition×hemisphere	0.02	0.901	0.00
Condition×location×hemisphere	1.59	0.219	0.06

**Familiar/unknown 500–800 msec**			
Condition	0.24	0.630	0.01
Condition×location	2.08	0.163	0.08
Condition×hemisphere	0.02	0.885	0.00
Condition×location×hemisphere	1.75	0.199	0.07

**Identify/familiar 300–500 msec**			
Condition	3.77	0.064	0.14
Condition×location	0.83	0.775	< 0.01
Condition×hemisphere	3.50	0.074	0.13
Condition×location×hemisphere	0.33	0.574	0.01

**Identify/familiar 500–800 msec**			
*Condition	11.32	0.003	0.33
*Condition×location	7.13	0.014	0.24
Condition×hemisphere	2.09	0.162	0.08
*Condition×location×hemisphere	7.81	0.010	0.25

## Footnotes

1 We also analyzed the data using a different ANOVA model, which incorporated midline electrodes into a hemisphere factor with three levels (left/midline/right). Both analysis schemes led to substantively the same outcome. We opted to report findings from the model with just two levels of the hemisphere factor (left/right) to reduce exposure to potential breaches of the sphericity assumption and for the clarity it affords in describing the findings.

2 Given that the old/new type effect is not reliable at the electrode where the difference is maximal, the interaction between condition and hemisphere appears to be due to contrasting patterns in the data across hemispheres rather than the presence of an effect on one hemisphere only. Close inspection of Fig. 1 shows a relatively more positive-going waveform for *familiar* compared to *unknown* on the left-hemisphere and the opposite pattern on the right-hemisphere, where the *familiar* waveform is more negative-going.
